# The relationships between Big Five Personality dimensions, harmful psychoactive substance use and academic motivation among undergraduates in Nigeria

**DOI:** 10.1192/bjo.2021.628

**Published:** 2021-06-18

**Authors:** Busola Awolaran, Emmanuel Babalola, Peter Onifade

**Affiliations:** 1 Kent and Medway NHS and Social Care Partnership Trust; 2 Hywel Dda University Health Board; 3 Drug Addiction Treatment and Education and Research, Neuropsychiatric Hospital Aro Abeokuta

## Abstract

**Aims:**

The aim of this study was to determine the relationships between personality traits, stress perception, academic motivation and harmful use (use related harmful consequences) of alcohol, tobacco and cannabis among undergraduates in Southwestern Nigeria.

**Method:**

The study is a descriptive cross-sectional study conducted among students of randomly selected tertiary institutions in south western Nigeria. Ethical approval was obtained from the Research and Ethics Commitee of the Federal Neuropsychiatric Hospital Abeokuta Ogun State Nigeria. Permission to carry out the study was sought from the University authorities. A multi-stage cluster sampling selection of 850 respondents was done. Consenting students were administered socio-demographic questionnaire, WHO student's drug use questionnaire, the Big Five Personality Inventory (BFI-44), perceived stress scale-10 and academic motivation inventory.

**Result:**

Seven hundred and eighty one completed questionnaires were analysed yielding a response rate of 92%. There were 51% males and 49% females with a mean age of 23.3 years (SD = ±2.29), from monogamous family setting 591(75%) and high socio-economic class (65.8%). Of the respondents, 24.8% reported experience of use related harmful consequences such as engaging in quarrel or argument, unprotected sex and sex regretted the next day. There were significant associations between male gender (p=<0.001), urban residence (p = 0.028), polygamous family setting (p = 0.002), high socioeconomic status (p = 0.026) and use related harmful consequences.

Multiple logistic regression showed that the odds of experiencing harmful consequences was less than 1 for agreeableness (OR = 0.515, df = 1, p = <0.001) and openness (OR = 0.634, df = 1, p = <0.028) but greater than 1 for extraversion (OR = 1.525, df = 1, p = <0.036) personality dimensions. This implies that for a unit increase in agreeableness and openness scores, there were decreased odds (8.6% and 79% respectively) of experiencing harmful consequences while there was increased odd (86%) of experiencing harmful consequences from a unit increase in extraversion score.

Both binary and multiple regression analysis revealed that the odds of experiencing harmful consequences is greater than 1 for perceived stress score (OR = 1.079, p = <0.001) and less than 1 for academic motivation (OR = 0.975, p = <0.001). This means that perceived stress is positively associated with substance use and experience of harmful consequences while academic motivation is negatively associated with substance use and experience of harmful consequences

**Conclusion:**

There were associations between certain socio-demographic factors, personality dimensions, stress perception and academic motivation with substance use and experience of harmful consequences. Thus, clinicians and researchers should consider these factors when designing preventive and treatment strategies.

